# Cloning, expression and activity of ATP-binding protein in *Bacillus thuringiensi*s toxicity modulation against *Aedes aegypti*

**DOI:** 10.1186/s13071-019-3560-2

**Published:** 2019-06-25

**Authors:** Guo-hui Zhao, Jian-nan Liu, Xiao-hua Hu, Khadija Batool, Liang Jin, Chen-xu Wu, Juan Wu, Hong Chen, Xiao-yan Jiang, Zhao-hui Yang, Xian-hui Huang, En-jiong Huang, Xiao-Qiang Yu, Xiong Guan, Ling-ling Zhang

**Affiliations:** 1 State Key Laboratory of Ecological Pest Control for Fujian and Taiwan Crops & School of Life Science, Fujian Agriculture and Forestry University, Fuzhou, 350002 China; 2 Fujian International Travel HealthCare Center, Fuzhou, 350001 China; 3 Guangzhou Key Laboratory of Insect Development Regulation and Application Research, Institute of Insect Science and Technology & School of Life Sciences, South China Normal University, Guangzhou, 510631 China; 4 Division of Cell Biology and Biophysics, University of Missouri-Kansas City, Kansas City, MO 64110 USA

**Keywords:** *Aedes aegypti*, ATP-binding protein, Biocontrol, Bti, Interaction

## Abstract

**Background:**

*Bacillus thuringiensi*s *israelensis* (Bti) is a widely used mosquitocidal microbial pesticide due to its high toxicity. ATP-binding proteins (ABP) are prevalently detected in insects and are related to reaction against Bti toxins. However, the function of ABP in mosquito biocontrol is little known, especially in *Aedes aegypti*. Therefore, this study aimed to clarify the function of ABP in *Ae. aegypti* against Bti toxin.

**Results:**

*Aedes aegypti* ABP (GenBank: XM_001661856.2) was cloned, expressed and purified in this study. Far-western blotting and ELISA were also carried out to confirm the interaction between ABP and Cry11Aa. A bioassay of Cry11Aa was performed both in the presence and absence of ABP, which showed that the mortality of *Ae. aegypti* is increased with an increase in ABP.

**Conclusions:**

Our results suggest that ABP in *Ae. aegypti* can modulate the toxicity of Cry11Aa toxin to mosquitoes by binding to Bti toxin. This could not only enrich the mechanism of Bt toxin, but also provide more data for the biocontrol of this transmission vector.

## Background

Mosquitoes, such as *Anopheles*, *Aedes* and *Culex*, are important infectious medium in transmitting various tropical diseases. Global health problems associated with mosquito-borne diseases put hundreds of millions of people at risk [1–3]. For example, *Aedes* spp. are the causative agent for dengue hemorrhagic fever and Zika virus [4, 5]. The attempt to control and early detection of these infections becomes a global public health issue [4]. However, there are no valid vaccines or medicines available presently. Vector control is the main method for preventing the spread of these diseases [2, 6, 7].

Presently, mosquitoes are mostly controlled by chemical pesticides. However, traditional chemical pesticides can cause environmental pollution and human health problems. Furthermore, it is easy for insect resistance to develop [8]. Nowadays, biological pesticides, in particular microbiological pesticides, are increasingly promoted due to being more environmentally friendly and having higher specificity, less influence on non-target organisms and inducing less resistance. Hence, microbiocontrol is regarded as one of the best means of control, and the application of entomopathogens to control mosquito populations is particularly effective [9].

*Bacillus thuringiensis* (Bt) is a Gram-positive bacterium used worldwide in biological control due to its high specificity to insects and low impact on the environment [10]. It can produce some pore forming toxins (PFTs, including Cry toxin and Cyt toxin) in its sporulation phase, which play an important role in the process of killing insects [11, 12]. They are toxic to more than 3000 species of insect in different orders, including Lepidoptera, Coleoptera and Diptera, etc. *Bacillus thuringiensis israelensis* (Bti) is widely used for its high toxicity against mosquitoes. It can produce a composite mosquitocidal crystal protein containing Cry2, Cry4, Cry10, Cry11, Cyt1 and Cty2 [13–15]. Among them, Cry11Aa can be activated by hydrolysis with proteinase in the mosquito midgut, and displays high toxicity to larvae of *Aedes* and *Culex*, but low toxicity against *Anopheles gambiae* [9, 15]. Once mosquito larvae have been exposed to the toxin, the activated toxin can bind to specific receptors to form an oligomer in the brush border membrane vesicles (BBMV) of the midgut [16–18], including the alkaline phosphatase (ALP), aminopeptidase N (APN), cadherin and ATP-binding cassette (ABC) transporters [9]. Oligomerization of toxins and insertion of toxin oligomers into the midgut epithelial cells cause perforation and cell death [16, 18]. However, some details in this process are still unclear, especially about the interaction of toxin and receptor [19]. Recently, the resistance of Bt toxin, which can diminish the toxicity in many insects, has drawn the public eye. As reported with organophosphate insecticides, some third-part proteins besides toxins and receptors can also be related to the insect resistance [8]. Because of the similar binding site, galectin could make *Caenorhabditis elegans* resistant to Cry5Ba toxin by binding to the receptors such as lipids and glycolipids [20]. In our previous work, we found that galectin-14 of *Aedes aegypti* can compete with Cry11Aa by binding to some of the Cry receptors, such as ALP1 [21]. Galectin-6 was also found to interact with ALP1 to affect the toxicity of Cry (unpublished results). C-type lectin can help *Anopheles gambiae* prevent *Plasmodium* infection *via* humoral immunity [22]. C-type lectin-20 was also found to interact with ALP1 to reduce Cry toxicity in *Ae. aegypti* [23]. Resistance can also be induced by the mutation of ATP-binding cassette (ABC) transporter in *Heliothis virescens* [24–28].

ABP is a large group of proteins that can hydrolyze ATP to provide energy for the transmembrane transport process. Recently, ABP was reported to play a role in the pathology of insects reacting to Bt toxin. A single amino acid mutation in ABP was found to improve the resistance of *Bombyx mori* against Bt toxin [29]. After an amino acid was inserted in ABP C2, the modified gene was expressed in the Sf9 cell. Bioassays showed that the modified ABP was less sensitive to Cry1A toxin in *B. mori* than the control ABP without modification [30]. After ABP G was silenced by RNAi, *Plutella xylostella* was found to be less sensible to the Cry toxin. As peroxidase C was found to be a Cry1Ab-binding protein in *Spodoptera exigua* [19], ABP in *Cx. quinquefasciatus* was also found to change the toxicity of Cry11Aa by interacting with this toxin [31]. These results all show that ABP might relate to the mechanism of Cry toxin against insects.

However, the function of ABP in mosquito biocontrol is still unclear, especially in *Ae. aegypti*. The aim of the present study was to clarify the function of ABP in *Ae. aegypti* against Bti toxin.

## Methods

### Mosquitoes, bacterial strains, antibodies and plasmids

*Aedes aegypti* Haikou strain was supplied by the Fujian International Travel HealthCare Center and maintained in our laboratory in an environment-controlled room at approximately 28 °C and 85% RH with a photoperiod of 14 h light and 10 h dark. The Cry11Aa recombinant Bt strain was kindly provided by Dr Sarjeet Gill, University of California, Riverside, CA, USA. *Escherichia coli* JM109 and BL21 were preserved in the Key Laboratory of Ecological Pest Control for Fujian and Taiwan Crops, Fujian Agriculture and Forestry University, and cultivated in Luria–Bertani (LB) liquid medium (10 g/l tryptone, 5 g/l yeast extract, 10 g/l NaCl, pH 7.2). Bt LLP29 was isolated and preserved in our previous work [32]. The rabbit polyclonal antibody against biotin, goat-anti-rabbit-AP-conjugated polyclonal antibody and the streptavidin horse-radish peroxidase (HRP) conjugate were purchased from Beyotime (Shanghai, China). The polyclonal antibody against Cry11Aa was produced in rabbits using purified recombinant Cry11Aa as an antigen [31]. The pMD18-T and pET-32a plasmids were purchased from TaKaRa (Dalian, China) and Novogene (Beijing, China), respectively.

### Acquisition of the ABP gene

Total RNA was extracted from *Ae. aegypti* using an E.Z.N.A.^®^ Total RNA Kit I (Omega Norcross, GA, USA), then reverse-transcribed using a PrimeScript^®^1stStrand cDNA Synthesis Kit (TaKaRa, Dalian, China), both according to the respective manufacturer’s instructions. The *ABP* gene (XM_001661856.2) was amplified by polymerase chain reaction (PCR) with specific primers designed according to the gene sequence in the National Center for Biotechnology Information (NCBI) database as follows: ATPF: 5′-GGA ATT CCA TAT GCA AAA TAA AGT GGT AAC CCT CAA AAC G-3′(*Nde*I restriction site underlined), ATPR: 5′-ACG CGT CGA CGC TCA CAT ACG GAT TGA TGT CCC GTT-3′ (*Sal*I restriction site underlined). *Nde*I/*Sal*I enzyme sites were selected for restriction digestion. The PCR product was purified using a DNA Gel Extraction Kit (Omega), and ABP gene was sequenced by Sangon Biotech (Shanghai, China).

### Cloning, expression and purification of ABP

In order to clone the ABP gene in *Ae. aegypti*, the purified PCR product was ligated with pMD-18T and then transformed into *E. coli* JM109 as described [31, 33]. Recombinant plasmid was digested with *Nde*I/*Sal*I and then ligated into the Pet-32 (+) expression vector [31, 33]. The PCR product of ABP from the recombinant plasmid was also sequenced by Sangon Biotech. The recombinant ABP was transformed into competent *E. coli* BL21 cells to express recombinant ABP. A blank Pet-32 (+) plasmid was also transformed into competent *E. coli* BL21 to express thioredoxin, which was used as a negative control in the subsequent experiments. After being induced by 0.5–1 mM isopropyl-β-d-1-thiogalactopyranoside (IPTG), the target protein was purified using a Protein ISO Ni-NTA Resin reagent kit (TransGen Biotech,Beijing, China) and then further labeled using EZ-Link-NHS-Biotin (Thermo Fisher Scientific, Waltham, MA, USA), both according the respective manufacturer’s instructions. Then, sodium dodecyl sulfate polyacrylamide gel electrophoresis (SDS-PAGE) was carried out to check the recombinant purified ABP [33]. Thioredoxin was also extracted using the same method.

### Cry11Aa protein preparation

The preparation of Cry11Aa protein from the recombinant Bt strain was carried out using the method described by Chen et al. [33–35]. The polyclonal antibodies against Cry11Aa protein were produced in rabbits using purified recombinant Cry11Aa. The secondary antibody goat-anti-rabbit-AP-labeled was detected using a BCIP/NBT alkaline phosphatase assay kit (Beyotime, Nanjing China).

### Western blot

In order to detect the recombinant proteins, western blot was carried out as follows. The biotinylated ABP and purified Cry11Aa were separated by 10% SDS-PAGE and transferred to a polyvinylidene fluoride (PVDF) membrane, which was further blocked with PBSM (PBS + 0.05% skim milk) for 2 h at 37 °C, and washed with PBST (PBS + 0.05% Tween-20). The membrane was then detected by the primary rabbit polyclonal antibody to biotin for ABP (1:3000) or the primary rabbit polyclonal antibody (1:3000) for Cry11Aa. The goat-anti-rabbit-AP-conjugated polyclonal antibody (1:3000) was used as the secondary antibody. All antibodies were diluted by PBS with 1% skim milk. A BCIP/NBT alkaline phosphatase assay kit was used to visualize the result, following the manufacturer’s instructions [14, 34, 35].

### Far-western blot

Far-western blot was used to detect the binding characterization between ABP and Cry11Aa protein. The biotinylated ABP and Cry11Aa were separated by 10% SDS-PAGE and transferred to a PVDF membrane. After being blocked with 5% dry skim milk in PBS for 2 h at 37 °C, the membrane with biotinylated ABP was then probed with the purified Cry11Aa protein (4 ng/ml, dissolved in PBS (pH 7.4) containing 0.1% BSA) overnight at 4 °C with gentle rocking, and then washed three times with PBST. The membrane was then incubated with primary rabbit polyclonal antibody to Cry11Aa (1:3000) and goat-anti-rabbit-AP-conjugated polyclonal antibody (1:3000) was used to detect Cry11Aa. After washing 3 times with PBST and PBS buffer, the binding of the interacting protein was checked by a BCIP/NBT alkaline phosphatase assay kit.

At the same time, the membrane with Cry11Aa was also incubated by biotinylated ABP (4 ng/ml), following probed by the primary rabbit polyclonal antibody (1:3000) to biotin and the secondary goat-anti-rabbit-AP-conjugated polyclonal antibody (1:3000). A BCIP/NBT alkaline phosphatase assay kit was used to visualize the binding protein following the manufacturer’s instructions [23].

### Enzyme-linked immunosorbent assay (ELISA)

To further understand the interaction between ABP and Cry11Aa, the purified recombinant ABP, Cry11Aa and thioredoxin were biotinylated with EZ-Link-NHS-Biotin following the manufacturer’s instructions. ELISA was then carried out as follows. Four micrograms of ABP or Cry11Aa was coated with ELISA buffer (Na_3_CO_3_, pH 9.6) in 96-well plate overnight at 4 °C, and then washed 3 times with PBS. ELISA buffer was only added to uncoated wells as a negative control. The increased concentration of Cry11Aa, biotinylated ABP or the control biotinylated thioredoxin (0 to 1280 nM) in 100 μl PBST were supplied to the protein-coated wells and further incubated for 2 h at 37 °C. The plates were then washed 3 times with 100 μl of PBST, and the bound biotinylated Cry11Aa, biotinylated ABP or thioredoxin were detected by incubation of the plates with the streptavidin horse-radish peroxidase (HRP) conjugate antibody (1:3000) for 2 h. After washing with 100 μl of PBST for 3 times, the chromogenic regent kit EL-TMB P0209 (Beyotime BiotechNanjing China) was used for the development of color. Absorbance was checked at 450 nm on a Multiskan™ GO Microplate Spectrophotometer (Thermo Fisher Scientific). Each treatment was repeated 3 times and data were analyzed using GraphPad Prism v.6.

### Bioassays

For bioassays with the purified ABP and Cry11Aa, third-instar *Ae. aegypti* larvae were fed with purified Cry11Aa protein (2.0 μg/ml) or a mixture of the purified Cry11Aa protein (2.0 μg/ml) and ABP (0, 1.0, 2.0, 3.0 or 4.0 μg/ml) in 30 ml of dechlorinated water. Thioredoxin was used as the negative control. Each treatment was replicated 3 times. The survival rates of the mosquito larvae were recorded after 12, 24 and 48 h [36]. The data of bioassays were further analyzed by IBM SPSS Statistics v.19.0.

## Results

### Cloning the ABP gene

**Fig. 1:**
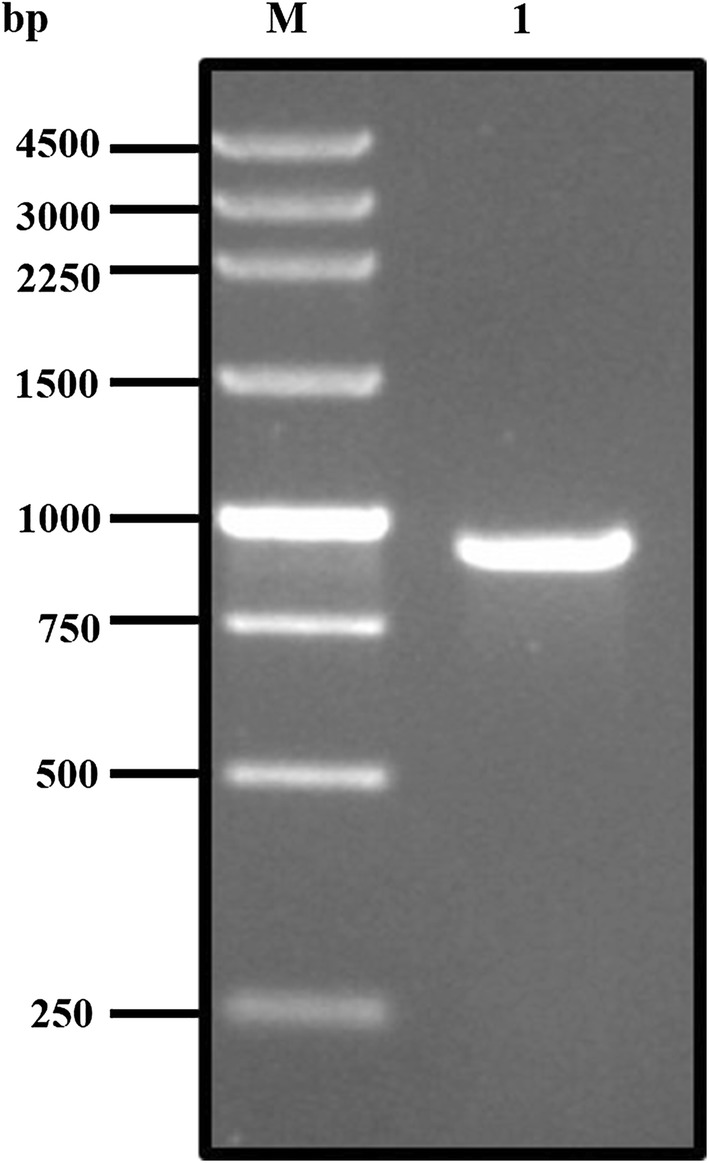
PCR product of ATP-binding protein. Lane M: 100 bp plus DNA ladder; Lane 1: PCR product of ATP-binding protein

**Fig. 2:**
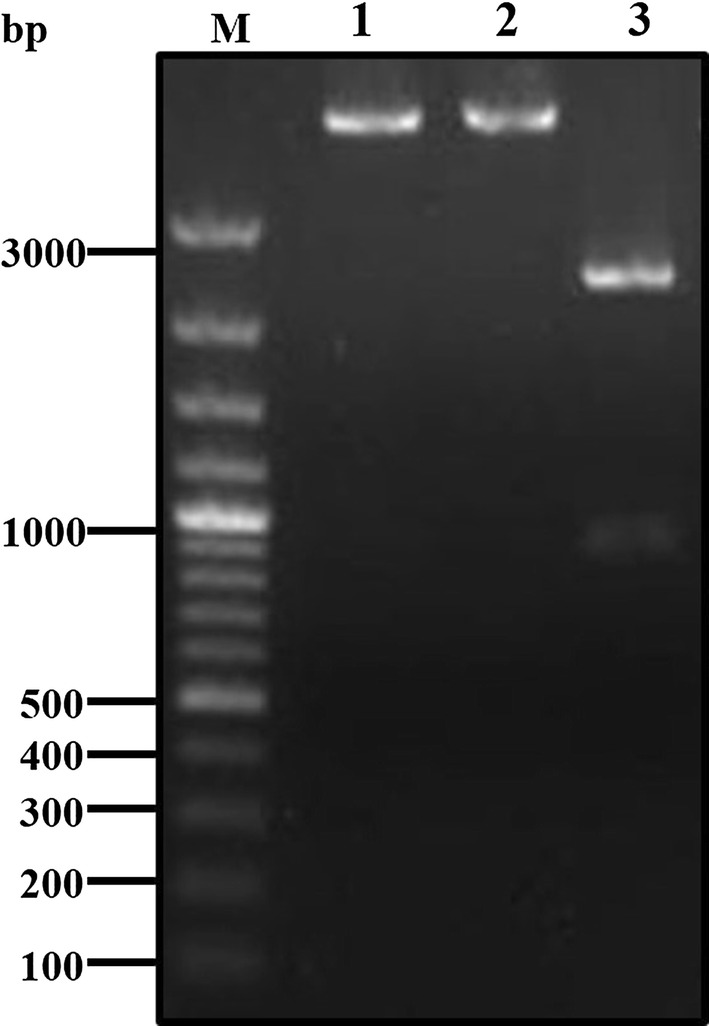
Restriction enzyme digestion of pMD-atp recombinant plasmid. Lane M: 100 bp plus DNA ladder; Lane 1: *Nde*I digested pMD-atp recombinant plasmid; Lane 2: *Sal*I digested pMD-atp recombinant plasmid; Lane 3: *Nde*I and *Sal*I simultaneously-digested pMD-atp recombinant plasmid

The PCR result showed that the full length ABP gene (GenBank: XM_001661856.2) has an open reading frame (ORF) of 915 bp encoding 304 amino acids (Fig. 1). The product was then purified and ligated into the cloning vector pMD-18T plasmid. After being transformed to JM109, positive colonies were obtained, and the sequence was confirmed by Sangon Biotech. The target fragment was also detected after the recombinant plasmid was digested by *Nde*I and *Sal*I restriction endonuclease (Fig. 2). The plasmid was then sequenced and the result showed a high homology (up to 99%) with the initial ATP-binding protein gene. All of these results show that the gene of the ATP-binding protein was successfully cloned.

### Expression and detection of ABP and Cry11Aa

**Fig. 3:**
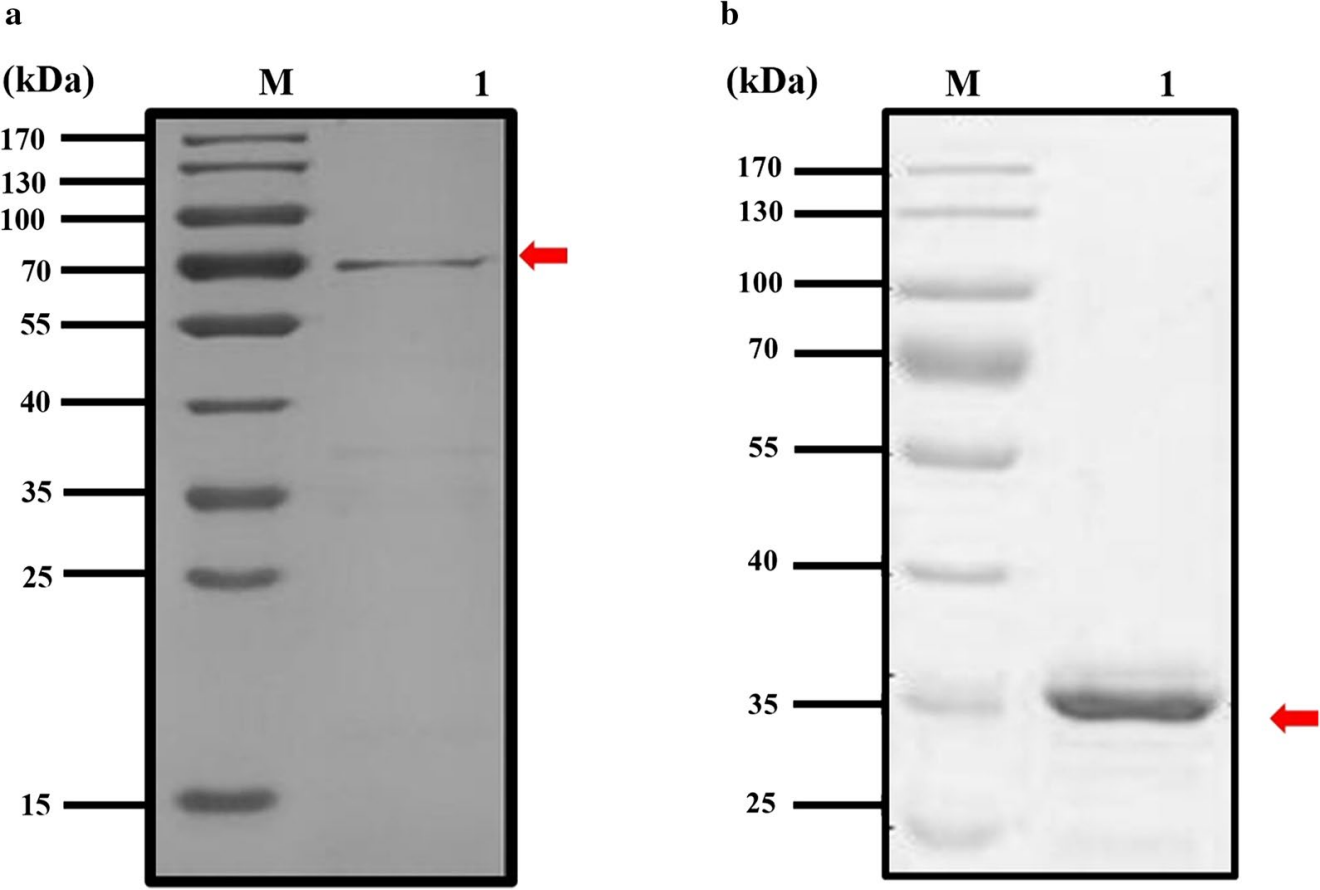
SDS-PAGE analysis purified Cry11Aa and ATP-binding protein. **a** SDS-PAGE analysis of the expressed protein (Lane M: protein molecular weight markers; Lane 1: purified Cry11Aa). **b** SDS-PAGE analysis of the expressed protein (Lane M: protein molecular weight markers; Lane 1: purified ATP-binding protein)

**Fig. 4:**
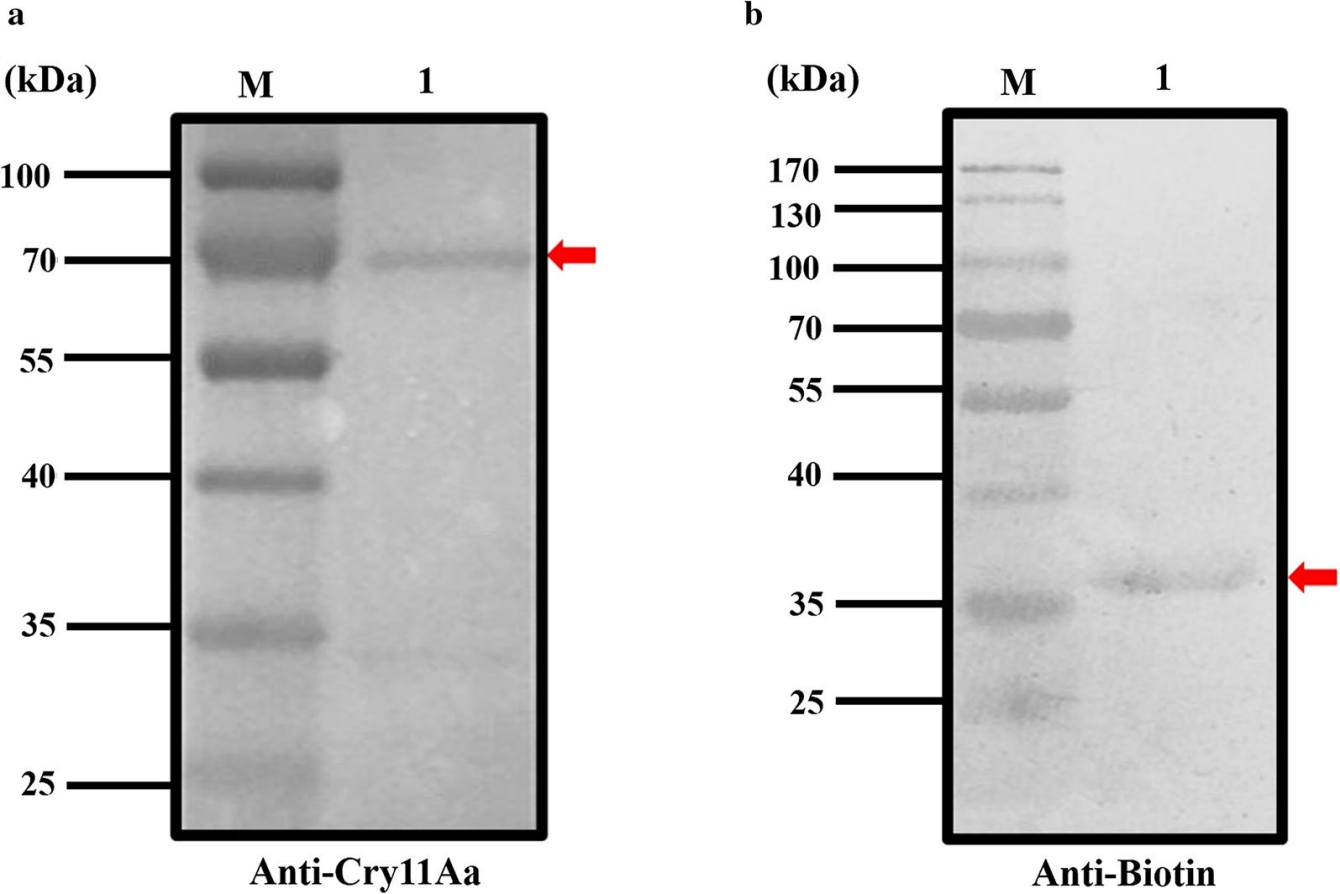
Western Blot analysis of Cry 11Aa and ATP-binding protein. **a** Western blot analysis of the expressed protein (Lane M: protein molecular weight markers; Lane 1: purified Cry11Aa). **b** Western blot analysis of the expressed protein (Lane M: protein molecular weight markers; Lane 1: purified biotinylated ATP-binding protein)

Cry11Aa crystal was expressed and purified from the recombined Bt strain that showed a 72 kDa band (Fig. 3a) in SDS-PAGE. Western blot also showed the 72 kDa band detected by the Cry11Aa specific antibody. From this, it can be concluded that Cry11Aa was expressed successfully (Fig. 4a). Meanwhile, the ABP gene was expressed in a prokaryotic expression system by ligating with the PET-32a expression vector. The PET-32a vector plasmid was digested with *Nde*I and *Sal*I prior to ligation with digested ABP gene from the ABP-pMD-18T recombinant plasmid. After the recombinant plasmid was transformed into *E. coli* BL21, the positive clones that were confirmed by PCR and enzyme digestion were selected. The sequence was also confirmed by Sangon Biotech. After being induced by 0.5–1 mM IPTG, the expressed ABP was purified using a Protein ISO Ni-NTA Resin reagent kit and labeled with EZ-Link-NHS-Biotin. The result of SDS-PAGE showed a 35 kDa band (Fig. 3b). The western blot also showed that ABP with a 35 kDa band was also detected by rabbit polyclonal antibody against biotin (Fig. 4b).

### Effect on toxicity of Cry11Aa by ABP

**Fig. 5:**
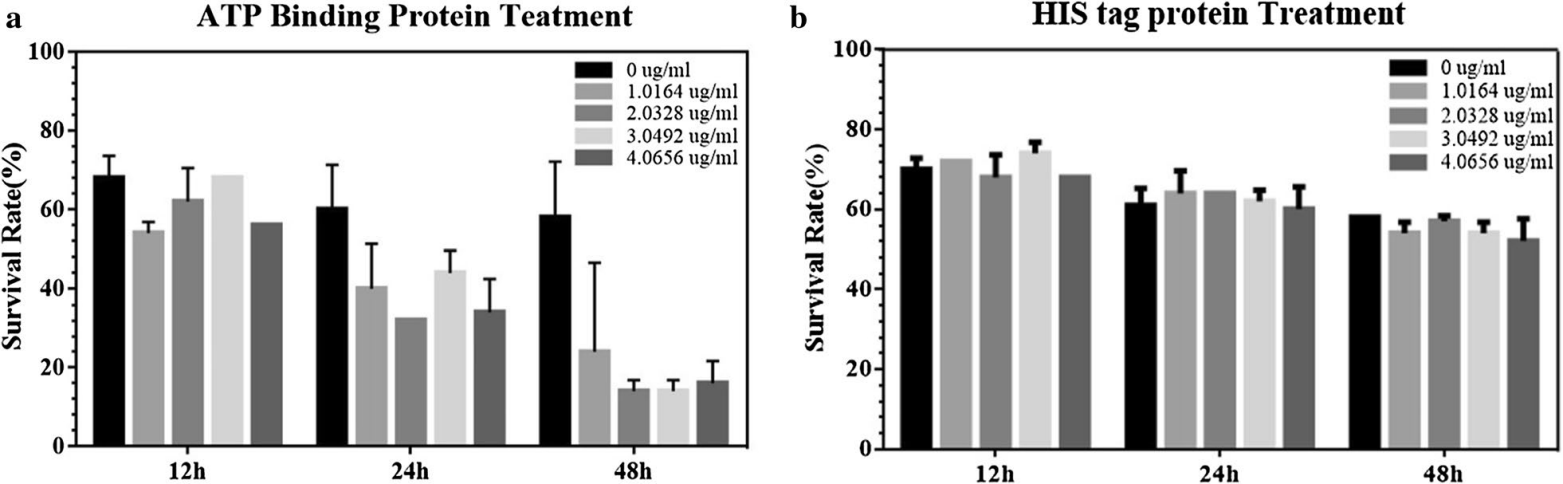
Bioassay of the ATP-binding protein. Each column represents the mean ± SEM (*n* ≥ 3). Each bar represents the mean ± SD of three technical replicates

In order to estimate the function of ABP in Cry11Aa toxicity, a bioassay against *Ae. aegypti* was carried out. The survival rate against Cry11Aa was recorded after 12, 24 and 48 h with the presence and absence of ABP. Results showed an obvious drop in the survival rate with increasing concentrations of ABP (Fig. 5); from this it can be deduced that ABP might enhance the toxicity of Cry11Aa toxin.

### Interaction of ABP and Cry11Aa

**Fig. 6:**
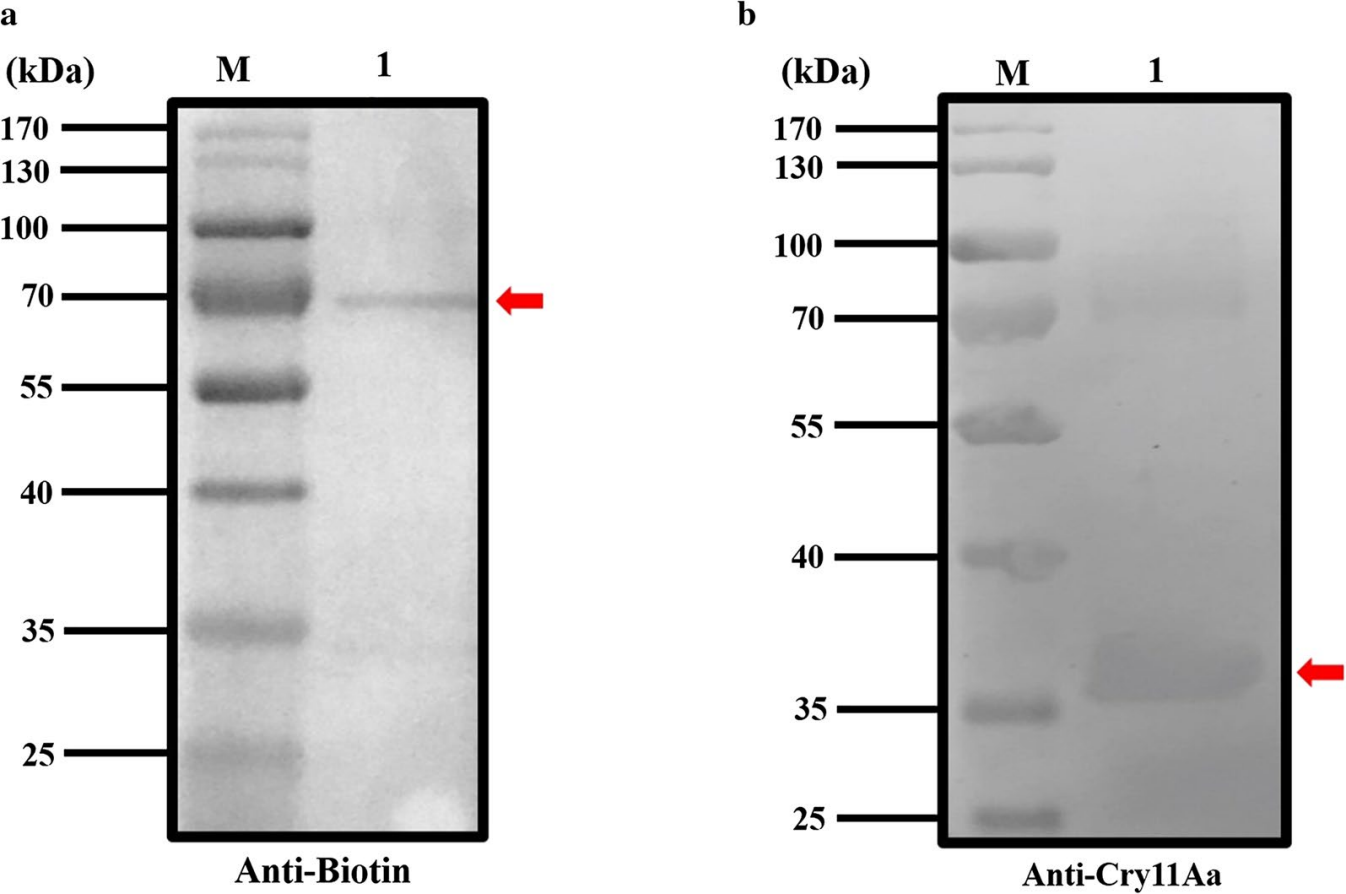
Far-western blot analysis of Cry 11Aa and ATP-binding protein. **a** Far-western blot analysis (Lane M: protein molecular weight markers; Lane 1: purified Cry11Aa probed with biotinylated ATP-binding protein). **b** Far-western blot analysis (Lane M: protein molecular weight markers; Lane 1: purified ATP-binding protein probed with Cry11Aa)

To test whether ABP could bind with Cry11Aa, the present study used western blot and far-western blot. After Cry11Aa was separated by SDS-PAGE and incubated with biotinylated ABP, the target band of 72 kDa was detected by specific rabbit polyclonal antibody against biotin (Fig. 6a); no band was detected after Cry11Aa was probed with the negative control thioredoxin. Similarly, when ABP was incubated with Cry11Aa, a band of 35 kDa could be detected by the specific antibody against Cry11Aa (Fig. 6b). These results show that ABP and Cry11Aa could bind to each other.

**Fig. 7:**
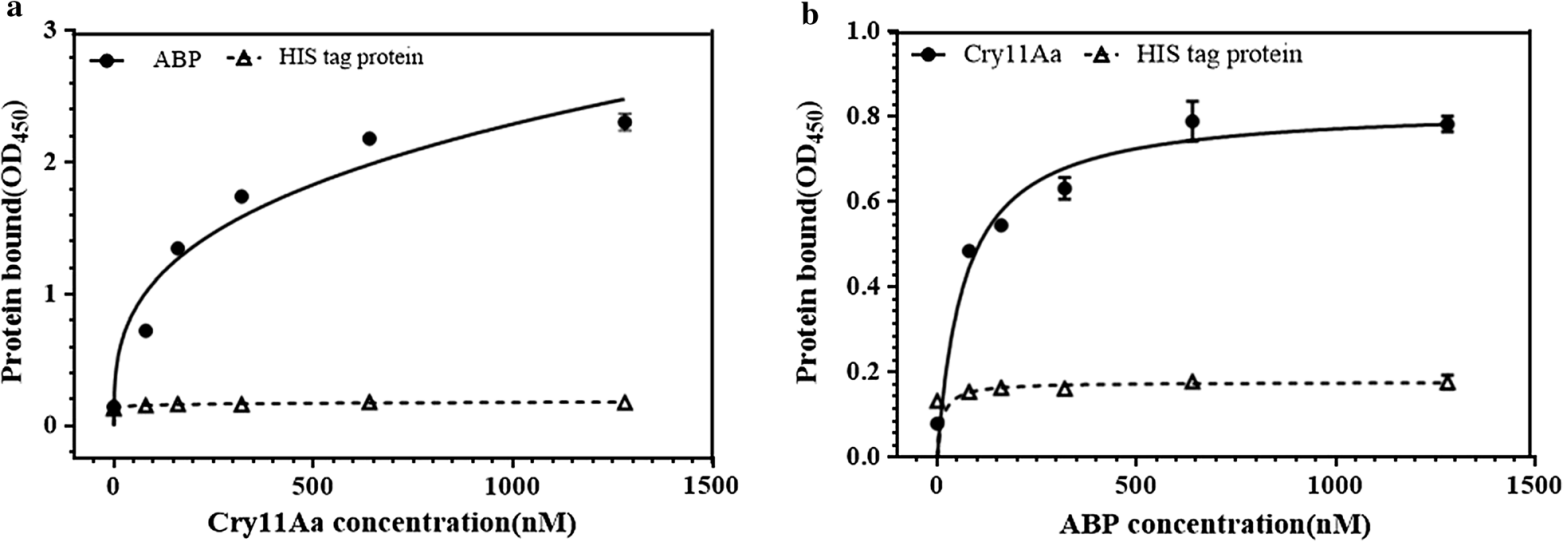
ELISA analysis between Cry11Aa and ATP-binding protein. **a** Total binding of ABP to Cry11Aa and **b** total binding of Cry11Aa binding to ABP. Each bar represents the mean ± SD of three technical replicates

ELISA was also carried out to further confirm the binding interaction between Cry11Aa and ABP. With an increasing concentration of biotinylated ABP used, more biotinylated ABP was found to bind with the immobilized Cry11Aa. The highest affinity was detected at a concentration of ABP of 1280 nM (Fig. 7a). Similarly, more Cry11Aa also bound to the immobilized ABP with an increased concentration of Cry11Aa and the binding condition was saturated when the concentration of Cry11Aa was up to 640 mM (Fig. 7b). In the negative control, both Cry11Aa and ABP were unable to bind with thioredoxin (Fig. 7). All of these results demonstrate that ABP can interact with Cry11Aa.

## Discussion

ABP is a large group of proteins that can be a transmembrane transporter with the help of ATP hydrolyzation. A significant amount of research shows that ABP plays a vital role in species of Lepidoptera such as *Bombyx mori* [29, 30], *Plutella xylostella* [37] and *Helicoverpa armigera* [38]. It was even found to interact with Cry11Aa and affect the toxicity of Cry11Aa against *Cx. quinquefasciatus* in our previous study [33]. However, there is limited research about the function of ABP in *Aedes*. Therefore, identification of the relationship between ABP of *Aedes* and Cry11Aa toxin can greatly enrich the knowledge of Bt mechanism.

In the present study, the ABP gene of *Ae. aegypti* was cloned and expressed to analyze the relationship between ABP from *Ae. aegypti* and Cry11Aa toxin from Bti. Similar to *Cx. quinquefasciatus*, ABP in *Ae. aegypti* was found to bind with Cry11Aa toxin. The toxicity of Cry11Aa toxin can be enhanced by ABP through its interaction with this toxin [33]. Although the detailed mechanism of ABP in enhancing the toxicity of Cry11Aa needs further study, our results indicate that ABP can bind effectively to Cry11Aa protein, similar to ALP and APN [9]; the toxicity of Cry11Aa can be influenced by this binding characterization in *Ae. aegypti.* ABP may be a factor that can interact with the toxin to form a composite to strengthen the toxicity or influence the physiological process in *Ae. aegypti* [33]. In order to further understand its function, ABP can be further silenced. Some other proteins in the mosquito midgut could also be tested to see whether they can alter the toxicity of Bt toxins. Such results could not only greatly enrich the knowledge of Bt mechanism, but also contribute to the discovery of new toxin receptors in pests, which could provide more data for the creation of new bio-pesticides.

## Conclusions

Mosquitoes play an important role in many infectious diseases. ABP is detected in many insects (including mosquitoes) related to the process of the Bti toxin reaction, which is widely used in mosquito biocontrol. In order to clarify the function of ATP-binding protein in *Ae. aegypti* against Bti toxin, the ABP gene of *Ae. aegypti* (GenBank: XM_001661856.2) was cloned, expressed and purified. Furthermore, results of far-western blot and ELISA showed an interaction between ABP and Cry11Aa. Our bioassay also showed that the mortality of *Ae. aegypti* increased with more the ABP added. Our results suggest that ABP in *Ae. aegypti* can modulate the toxicity of Cry11Aa toxin to the mosquito by binding to the Bti toxin.

## Data Availability

Data supporting the conclusions of this article are included within the article. The raw data are available from the corresponding author upon request. The sequence of the ATP-binding protein used in this paper refers to the NCBI GenBank accession number XM_001661856.2.

## Abbreviations

ABC transporters: ATP-binding cassette transporters
Bt: *Bacillus thuringiensis*
Bti: *Bacillus thuringiensis israelensis*
ALP: alkaline phosphatase
APN: aminopeptidase-N
LB: Luria–Bertani
Trx: thioredoxin
PCR: polymerase chain reaction
SDS-PAGE: sodium dodecyl sulfate polyacrylamide gel electrophoresis
IPTG: isopropyl-β-d-1-thiogalactopyranoside
ELISA: enzyme-linked immunosorbent assay
PBS: phosphate-buffered saline
PBS-T: phosphate-buffered saline containing Tween-20
